# Frederic Mohs: A Trailblazer in Dermatologic Surgery

**DOI:** 10.7759/cureus.70850

**Published:** 2024-10-04

**Authors:** Calista Pappas, Michelle L Demory

**Affiliations:** 1 Medicine, Nova Southeastern University Dr. Kiran C. Patel College of Osteopathic Medicine, Fort Lauderdale, USA; 2 Microbiology and Immunology, Nova Southeastern University Dr. Kiran C. Patel College of Osteopathic Medicine, Fort Lauderdale, USA

**Keywords:** basal cell carcinoma, dermatologic surgery, dermatology, dermatopathology, frederic mohs, historical vignette, medical pioneer, melanoma, mohs surgery, squamous cell carcinoma

## Abstract

Frederic Mohs was an American physician and surgeon who revolutionized the treatment of skin cancer through the development of the Mohs micrographic surgery technique. Born in 1910, Mohs devised this innovative procedure while still a medical student, seeking a more effective method for removing skin cancers with minimal damage to surrounding healthy tissue. The technique, which involves the precise removal of cancerous tissue, layer by layer, while examining each under a microscope, allows for the highest possible cure rates and the preservation of as much healthy tissue as possible. Mohs' method has since become the gold standard in dermatologic surgery for certain types of skin cancer, significantly improving patient outcomes. His contributions have had a lasting impact on both the medical field and the lives of countless patients.

## Introduction and background

**Figure 1:**
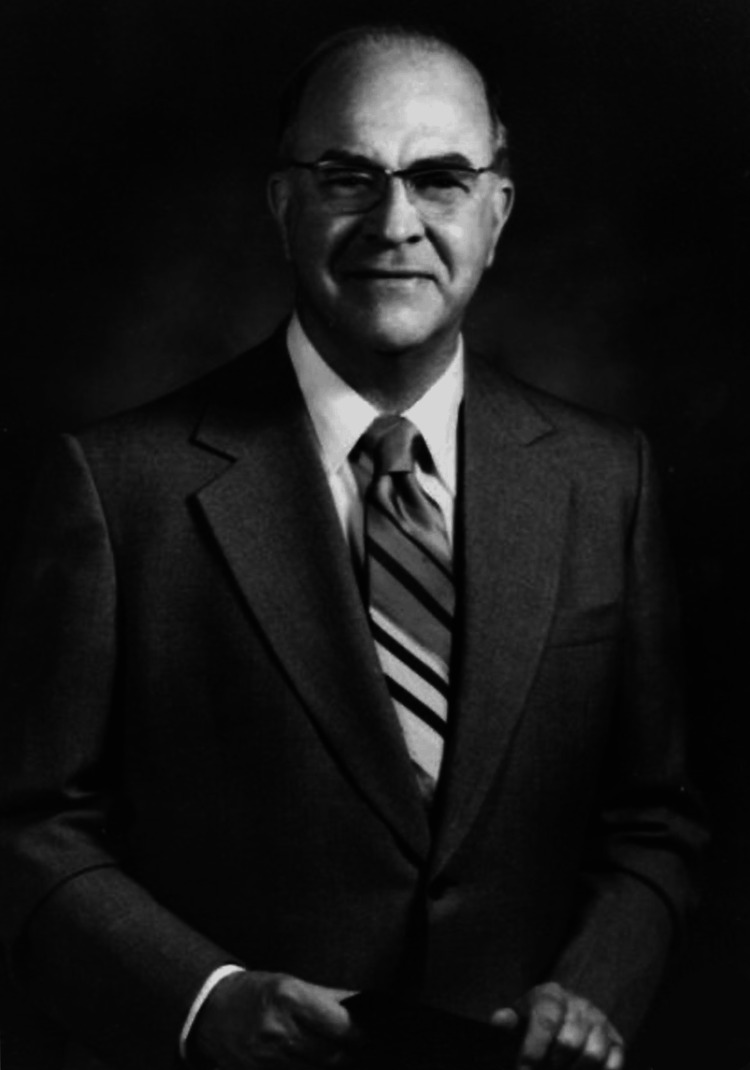
Frederic Edward Mohs, 1910-2002 Credit: Permission obtained from UW-Madison Archives & Records [2]

Frederic Edward Mohs, M.D., revolutionized the field of dermatology with the development of Mohs micrographic surgery (MMS), a precise technique for removing skin cancer. This innovative procedure has become the gold standard for treating various types of skin cancers, particularly basal cell carcinoma (BCC) and squamous cell carcinoma (SCC) [1-4]. Dr. Mohs' medical contributions extend beyond this surgical method, influencing cancer treatment protocols and improving patient outcomes worldwide [5-8]. This review explores Mohs' contributions to dermatology, the development and evolution of his technique, and its impact on contemporary practices (Figure 1).

## Review

Early life and education

Frederic Edward Mohs was born on March 1, 1910, in Burlington, Wisconsin, where he grew up in a small Midwestern town. From an early age, he developed a strong work ethic and a passion for science and medicine, setting the stage for his groundbreaking contributions to dermatology [8-12].

Mohs attended the University of Wisconsin for both his undergraduate and medical education. During his time as a student, he became deeply interested in cancer research, particularly under the mentorship of Dr. Michael Guyer, a zoologist and cancer researcher [13]. This early exposure to tumor biology significantly influenced Mohs' later work in developing a precise method for treating skin cancer.

In 1936, while still in medical school, Mohs conceived the initial concept for what would become known as Mohs surgery. His innovative approach involved using microscopic control to ensure the complete removal of cancerous tissue while preserving as much healthy tissue as possible. This technique was rooted in his early research and a deep understanding of cancer growth [14-16].

Dr. Mohs began his medical career focused on general surgery, but his interest in dermatology and skin cancer treatment grew over time. He became increasingly aware of the limitations of traditional surgical methods for skin cancer removal, which fueled his determination to improve surgical outcomes and reduce recurrence rates [17-21].

The foundation laid during Frederic Mohs' early life and education ultimately led to his pioneering work in dermatologic surgery, culminating in the development of the highly effective MMS technique.

Development of MMS

**Figure 2:**
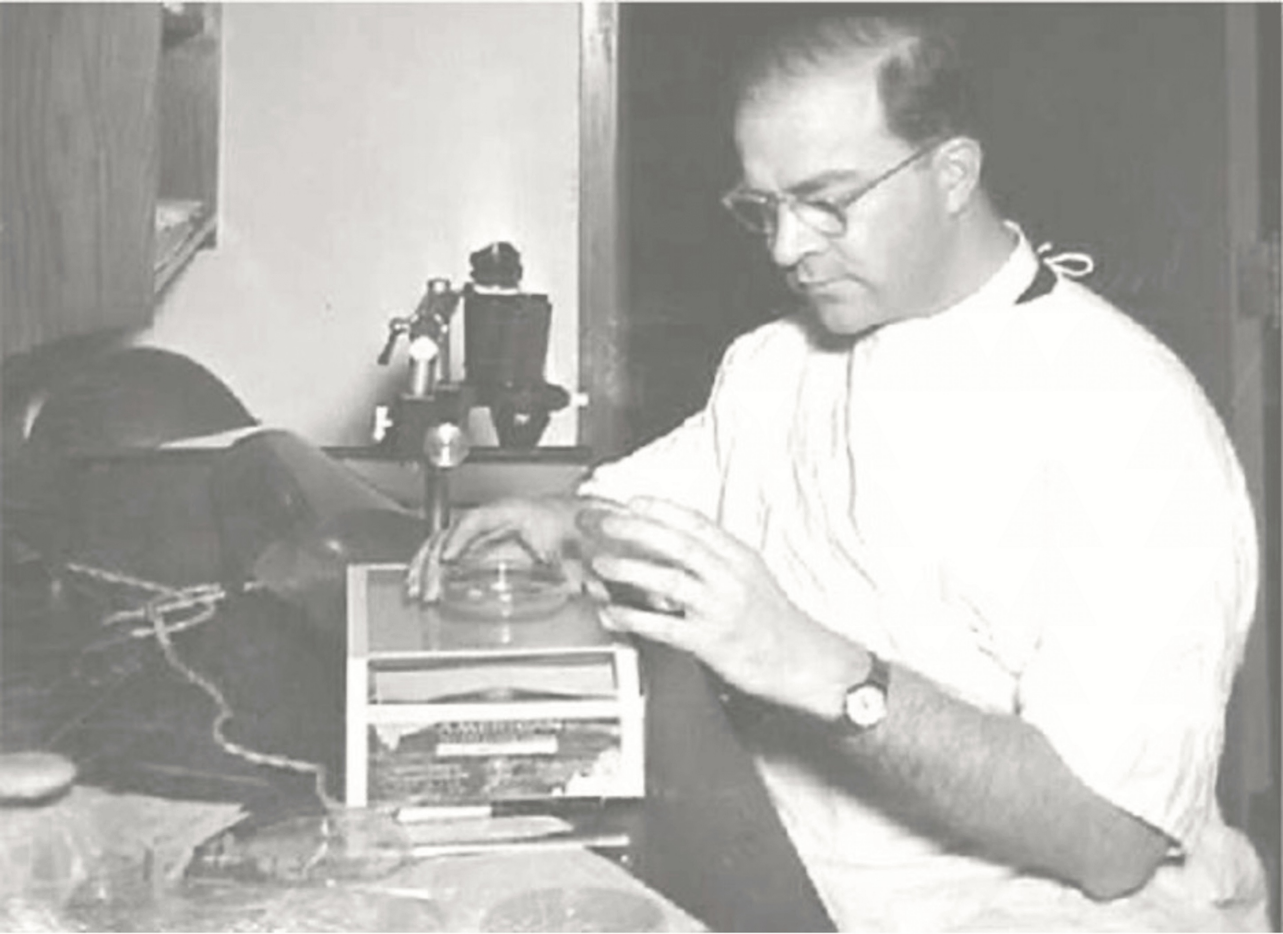
Dr. Mohs is pictured performing chemosurgery Credit: Permission obtained from UW-Madison Archives & Records [2]

During his residency at the University of Wisconsin in the late 1930s, Dr. Mohs began developing a groundbreaking technique that would eventually bear his name. Initially termed "chemosurgery," this method allowed for the precise excision of cancerous tissue while preserving as much healthy tissue as possible [4-7]. Over time, Dr. Mohs refined the technique by incorporating microscopic examination of the excised tissue, ensuring the complete removal of cancer cells (Figure 2).

The original Mohs procedure is based on the principle that skin cancers grow in a contiguous manner from a central origin. The procedure was designed to completely remove the tumor while preserving as much healthy tissue as possible [6-8]. Initially, the "chemosurgery" technique involved using a chemical fixative called zinc chloride paste, which was applied to the tumor in situ. This fixative allowed the tissue to be removed in thin layers over time without bleeding, and each layer was examined microscopically to check for cancer cells. The original Mohs procedure aimed to achieve clear margins by removing successive layers of tissue until no cancer cells were found [9].

The cornerstone of Dr. Mohs' legacy is the development of this procedure, which revolutionized skin cancer treatment. His method of layer-by-layer excision with microscopic examination significantly reduces the likelihood of cancer recurrence and minimizes the need for additional surgeries or radiation therapy. However, the original technique had some limitations, including the lengthy process and patient discomfort caused by the zinc chloride application [10-14]. To address these challenges, Dr. Mohs explored the use of "fresh tissue" instead of relying on the paste [11-13].

The first "fresh tissue" Mohs surgery was conducted on the periocular region, marking a significant milestone in dermatologic surgery [8-10]. Dr. Mohs administered local anesthesia, excised a thin layer of fresh tissue, and immediately examined it under a microscope. Upon finding tumor cells at the margins, he promptly removed another layer of tissue. The delicate nature of the eye and surrounding tissues required a method that could ensure complete cancer removal while preserving as much healthy tissue as possible to maintain function and appearance [8-9].

**Figure 3:**
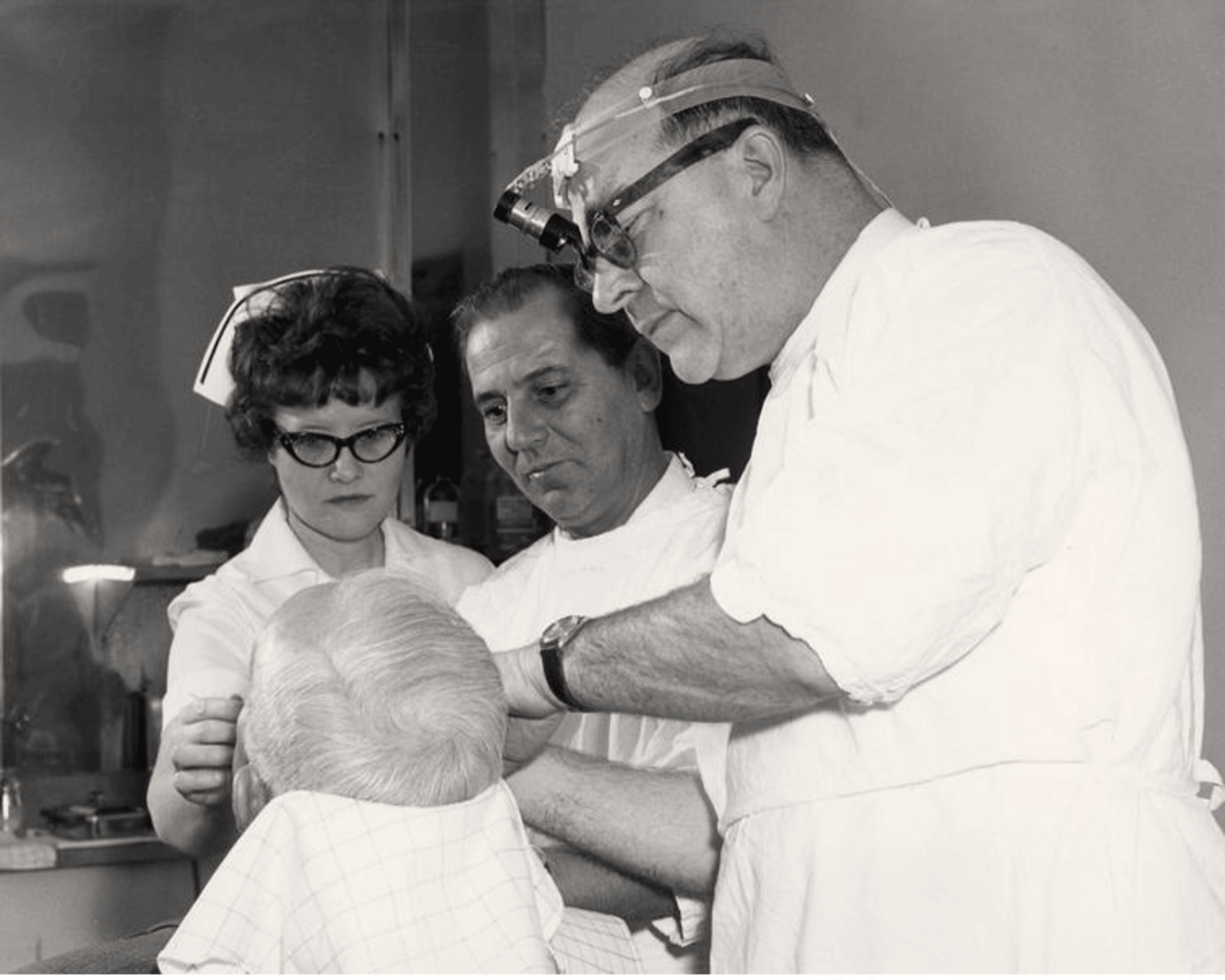
Dr. Mohs is pictured with clinic staff and patient, on whom he appears to be performing procedure Credit: Permission obtained from UW-Madison Archives & Records [2]

Recognizing the potential of his micrographic surgery technique for treating cancers in sensitive areas, Dr. Mohs began applying this method to the periocular region by the 1950s [5]. These early surgeries demonstrated that his technique could be effectively adapted to challenging cases, underscoring its precision and efficacy. The success of these procedures led to the widespread adoption of Mohs surgery for treating skin cancers in cosmetically and functionally critical areas, like the eyelids and surrounding tissue (Figure 3) [13-21].

Over time, the procedure evolved into what is now known as MMS, where the chemical fixation step was replaced with a fresh tissue technique. In this modern approach, tissue is immediately processed using frozen sections, enabling faster results and same-day completion of the surgery [10-14]. Despite these advancements, the core principles of the original Mohs procedure - tumor removal with complete microscopic margin examination - remain integral to the technique [11].

MMS involves systematically removing layers of skin cancer and examining each layer under a microscope until no cancer cells are detected. This meticulous process ensures that all cancerous tissue is removed while preserving as much healthy tissue as possible, leading to high cure rates and excellent cosmetic and prognostic outcomes [13-21].

Today, MMS is the preferred method for treating periocular skin cancers, offering the highest cure rates while minimizing damage to the delicate structures of the eye [15-16,20]. Over the years, advancements in imaging and surgical technologies have further enhanced the procedure, solidifying its status as the gold standard for treating certain types of skin cancer, particularly non-melanoma skin cancers like BCC and SCC [4].

Impact on dermatology and oncology

Dr. Mohs' technique has revolutionized skin cancer treatment. Mohs surgery achieves cure rates of up to 99% for primary BCC and SCC, making it one of the most effective treatments available [3-6]. The procedure is particularly valuable for tumors with poorly defined borders, recurrent cancers, and those located in cosmetically or functionally sensitive areas, such as the face, ears, and genitals [5].

The Mohs procedure was developed based on the principle that cutaneous malignancies spread outward from a central point, making complete tumor removal essential for achieving effective local control [13]. Over the years, MMS has developed into a fresh tissue technique using frozen sections. This modern method eliminates the need for in situ tissue fixation before excision, instead rapidly processing the tissue after excision by embedding it in a medium, freezing it with a cryostat, and sectioning it for histologic staining [3].

MMS is divided into two main phases: surgery and pathology. Techniques among Mohs surgeons can vary, particularly in tumor debulking, layer removal, and specimen marking [20]. However, all Mohs procedures share key elements: identifying tumor margins clinically, removing the visible tumor with 1-3 mm margins in a disc or saucer shape, marking the tumor bed to align the surgical site with the excised specimen, and mapping the specimen. During mapping, the tissue is sectioned, and the edges are dyed with various colors to distinguish individual margins, which are then matched to the corresponding areas on the tissue map [17].

The success of Mohs surgery has led to its widespread adoption and the establishment of specialized training programs. Dermatologists and surgeons undergo rigorous training to master this technique. During a Mohs fellowship, dermatologists receive intensive training in various areas, including surgical techniques, pathology, and reconstructive surgery [5].

Mohs surgery is highly effective and unique due to its approach of microscopically examining 100% of the surgical margins. The Mohs surgeon, who is specially trained in both surgery and pathology, performs the on-site interpretation of the tissue margins [21]. This expertise allows for precise correlation of microscopic findings with the surgical site on the patient. Extensive experience is required in interpreting microscopic slides of skin tissue, ensuring the complete removal of cancerous cells while preserving healthy tissue. The procedure ensures complete cancer removal during surgery, significantly reducing the likelihood of the cancer returning. It also minimizes the removal of healthy tissue, thereby maximizing both functional and cosmetic outcomes. In many cases, the surgical site can be repaired on the same day the cancer is removed. Additionally, Mohs surgery has a high success rate in curing skin cancer, especially in cases where other treatments have failed [1].

Other skin cancer treatment methods often estimate the amount of tissue to remove, which can lead to unnecessary removal of healthy skin and increase the risk of tumor regrowth if any cancer is missed. While treating the cancer is the primary concern, reconstructing the treated area is also important. After confirming the complete removal of cancerous tissue, the Mohs surgeon will decide on the most suitable method for wound repair [17]. Fellowship-trained Mohs surgeons have specialized skills in reconstructive surgery, ensuring optimal results for both function and appearance. Their expertise in performing complex reconstructions is often necessary after the removal of skin cancer to restore the appearance and function of the affected area. Upon completion, fellows are well-equipped to perform Mohs surgery independently and are considered experts in the field of dermatologic oncology and surgery [1,5-6].

Originally developed for BCC, MMS is now used to treat a wide range of skin cancers, including SCC and certain types of melanoma [3]. The technique has also been adapted for various anatomical sites, such as the eyelids, ears, and nose. The widespread success of MMS has driven the establishment of specialized training programs for dermatologic surgeons, with an emphasis on precision, patient care, and ongoing research to continue advancing the field [21].

Frederic Mohs' contributions have had a transformative impact on dermatology and skin cancer treatment. The introduction of MMS has led to several significant advancements in the field, including improved patient outcomes and expanded indications for its use [1-4]. The high cure rates associated with MMS have greatly enhanced patient outcomes, particularly in the treatment of skin cancers in cosmetically sensitive areas, like the face, where minimal scarring and functional preservation are critical [4-6,12].

Legacy and recognition

**Figure 4:**
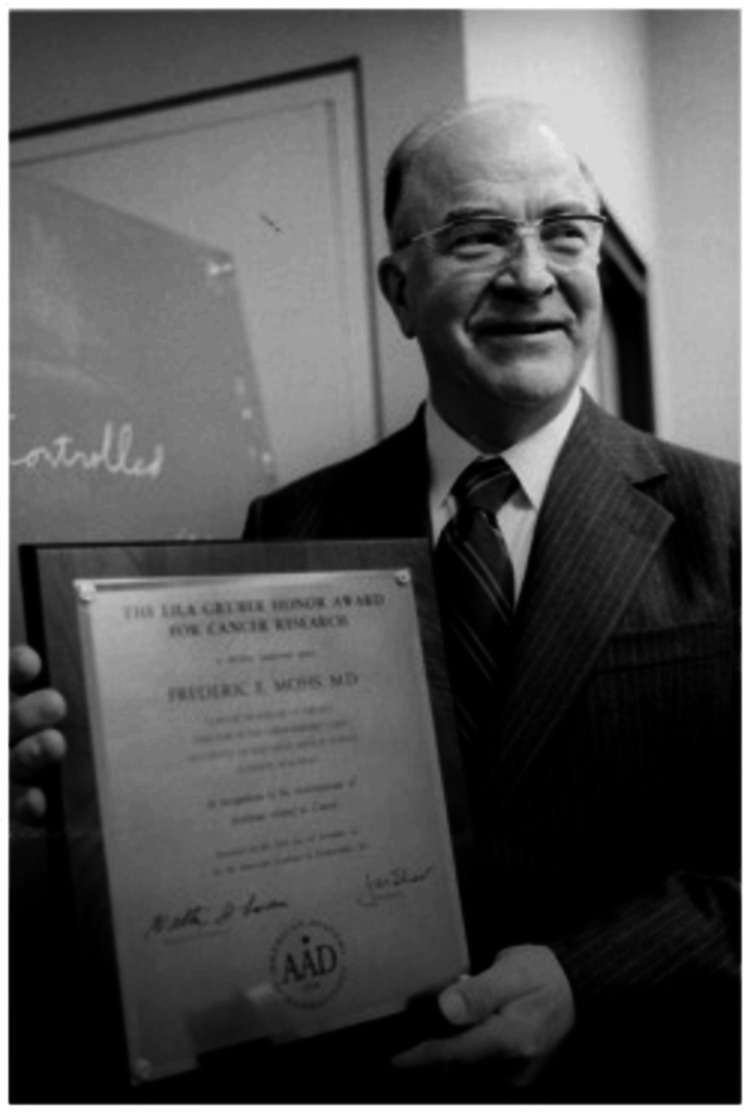
Dr. Mohs is pictured receiving an award from the American Academy of Dermatology Credit: Permission obtained from UW-Madison Archives & Records [2]

Frederic Mohs received numerous accolades throughout his career in recognition of his pioneering work in dermatologic surgery. His work was widely published, and he was frequently invited to speak at medical conferences and lectures, further cementing his reputation as a leader in dermatologic surgery [8]. Dr. Mohs' achievements in medicine were celebrated with several prestigious awards, including the American Academy of Dermatology's Lila Gruber Award for Cancer Research in 1977, the International Facial Plastic Surgery Award in 1979, the Frederic E. Mohs Award from the Skin Cancer Foundation in 1982, the Discovery Award of the Dermatology Foundation in 1995, and a commendation from the Office of the Governor of the State of Wisconsin in 1996 (Figure 4) [10].

Dr. Mohs' enduring legacy is celebrated through the Frederic E. Mohs Award, established by the American College of Mohs Surgery. This prestigious award honors individuals who have made significant contributions to dermatologic surgery, reflecting Dr. Mohs' lasting influence on the specialty [14-16].

Frederic Mohs left a lasting legacy in the field of dermatology through his development of MMS, a technique that revolutionized the treatment of skin cancer. His innovative approach has become the gold standard for treating many types of skin cancer, particularly BCC and SCC [17].

Dr. Mohs' legacy extends beyond the technique itself. His work has had a profound impact on patient care, offering higher cure rates, lower recurrence rates, and better cosmetic outcomes than many traditional methods [6]. Today, Mohs surgery is widely practiced around the world, with thousands of surgeons trained in this specialized technique. His commitment to both patient care and scientific advancement has set a high standard for future generations of dermatologists [20].

In addition to his contributions to skin cancer treatment, Mohs also played a key role in training and mentoring future surgeons. His influence is evident in the continued evolution of the procedure and the ongoing research in the field. The American College of Mohs Surgery, founded in his honor, continues to promote excellence in the practice and further advancements in skin cancer treatment [16]. Frederic Mohs' work transformed the field of dermatologic surgery, and his legacy continues to save lives and improve patient outcomes to this day.

## Conclusions

Frederic E. Mohs, M.D., was a trailblazer in dermatology whose innovative technique transformed skin cancer treatment. His development of MMS has saved countless lives and established a new benchmark for precision in cancer care. Dr. Mohs' lasting legacy is reflected in the continued use and refinement of his technique, ensuring that patients benefit from his pioneering work for generations.

Dr. Mohs' contributions to dermatology, particularly through the development of MMS, mark a significant leap forward in skin cancer treatment. His groundbreaking approach set a new standard for precision and patient care, leading to better outcomes and lower recurrence rates. As dermatologic surgery continues to advance, the principles laid down by Mohs will undoubtedly remain a foundational element of effective and compassionate skin cancer treatment.

## References

[1] (2024). Mohs micrographic surgery. https://www.aafp.org/pubs/afp/issues/2005/0901/p845.html.

[2] Dr. Frederic E (2024). UWDC digital collections. https://search.library.wisc.edu/search/digital?q=mohs.

[3] Weinzweig J (2024). Principles of Mohs surgery. Plastic Surgery Secrets Plus.

[4] (2024). History of Mohs micrographic surgery. https://link.springer.com/chapter/10.1007/978-3-031-52434-9_1.

[5] (2024). History of MOHS - the skin cancer foundation. https://www.skincancer.org/treatment-resources/mohs-surgery/history-of-mohs/.

[6] Mohs FE (1941). Chemosurgery: a microscopically controlled method of cancer excision. Arch Surg.

[7] Mohs FE (1980). Chemosurgery. Clin Plast Surg.

[8] Mohs FE (1989). Mohs micrographic surgery: a historical perspective. Dermatol Clin.

[9] (2024). American College of Mohs surgery. https://www.mohscollege.org/about-acms/history-of-mohs-surgery.

[10] (2024). History of Mohs surgery. http://www.mohssurgery.org/about-asms/about-mohs-surgery/history-mohs-surgery/.

[11] Sood A, Ayyaswami V, Prabhu AV, Benedek TG (2017). Frederic Edward Mohs, MD-the pioneer of chemosurgery. JAMA Dermatol.

[12] Frederic E. Mohs, M.D M.D (2024). Frederic E. Mohs, M.D. (1910-2002): physician and innovator. https://jdc.jefferson.edu/gibbonsocietyprofiles/43.

[13] Brodland DG, Amonette R, Hanke CW, Robins P (2000). The history and evolution of Mohs micrographic surgery. Dermatol Surg.

[14] DePaolo C (2018). Frederic E. Mohs, MD, and the history of zinc chloride. Clin Dermatol.

[15] Hanke CW (2002). Frederic E. Mohs, MD—the first Mohs micrographic surgeon: March 1, 1910-July 1, 2002. Dermatol Surg.

[16] Trost LB, Bailin PL (2011). History of Mohs surgery. Dermatol Clin.

[17] (2024). What is Mohs microscopic surgery?. https://theskinsurgerycentre.com/procedures/mohs-micrographic-surgery/.

[18] (2024). Frederic E. Mohs. https://en.wikipedia.org/wiki/Frederic_E._Mohs.

[19] Ross NA, Saedi N, Yeo CJ, Cowan S (2015). Frederic E. Mohs, M.D. (1910-2002): physician and innovator. Am Surg.

[20] Connolly K, Chow M, Afzalneia R, McKay C, Nehal KS (2024). History of Mohs micrographic surgery. Lab Man Mohs Micrographic Surg.

[21] Levy AL, Stasko T (2012). Mohs surgery. Evidence-Based Procedural Dermatology.

